# Ruptured Ovarian Artery Pseudoaneurysm in a Postmenopausal Patient Treated with Transcatheter Embolization

**DOI:** 10.1155/2020/6728318

**Published:** 2020-02-14

**Authors:** Michael M. Herskowitz, Chi Mei Wong, Robert F. Leonardo

**Affiliations:** Department of Radiology, SUNY, HSCB, 450 Clarkson Avenue, Brooklyn, NY 11215, USA

## Abstract

Ovarian artery pseudoaneurysms are relatively rare entities, usually associated with pregnancy or the early postpregnancy period. Occurrence in older females is even less common. We present a case of ruptured ovarian artery pseudoaneurysm in an older postmenopausal patient, successfully treated with transcatheter embolization.

## 1. Introduction

A rupture of an ovarian artery pseudoaneurysm is included in the differential diagnosis of a spontaneous atraumatic retroperitoneal hemorrhage. They are relatively uncommon entities, and the majority occur either late in pregnancy or in the early postpregnancy period. Such occurrences in postmenopausal patients are even less common, with fewer than ten cases reported in the world's literature.

Angiography with embolization has replaced open surgery as the treatment of choice for such lesions.

We present an additional case of spontaneous rupture of an ovarian artery pseudoaneurysm in a postmenopausal patient.

## 2. Case Presentation

**Figure 1:**
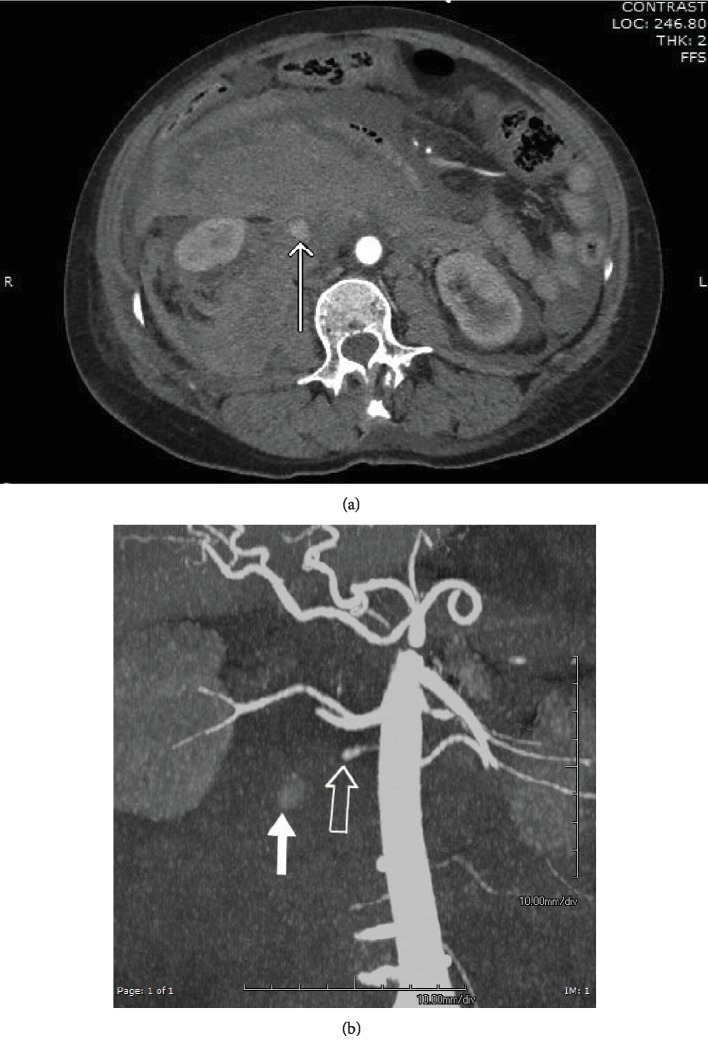
(a) Axial image from contrast-enhanced CT demonstrates collection of contrast suggestive of pseudoaneurysm (arrow) with surrounding massive retroperitoneal hemorrhage. (b) Coronal maximum intensity projection image from CT scan shows partial filling of vessel arising from the aorta presumed to be the right ovarian artery (open arrow) and collection of contrast suggestive of pseudoaneurysm (solid arrow).

A 59-yeart-old black female presented to the emergency department with acute onset of abdominal pain, hypotension, and severe anemia. There is a history of chronic lymphocytic leukemia, currently in remission as well as medically controlled hypertension. In the emergency room, laboratory values were remarkable for hemoglobin/hematocrit = 4.4/13.1, platelets = 192, WBC = 44.5, INR = 1.2, calcium = 6.8, albumin = 1.6, and BUN/creatinine = 51/2.2. The patient was immediately transfused 3 units packed RBCs, 2 liters crystalloid, and appropriate calcium infusion. A contrast-enhanced CT scan of the abdomen and pelvis was performed which demonstrated active extravasation of contrast in the right infrarenal retroperitoneal space (Figure 1(a)). CTA reconstructions suggested an origin from the right ovarian artery (Figure 1(b)).

**Figure 2:**
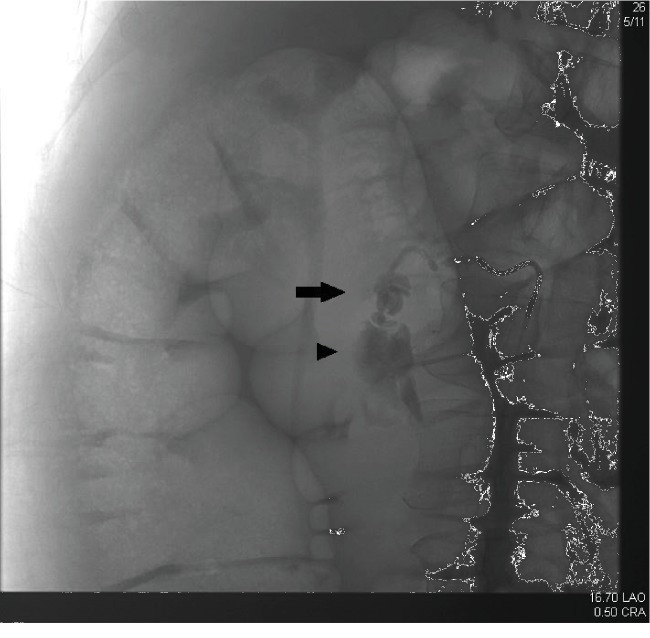
Right ovarian arteriogram demonstrating proximal pseudoaneurysm (arrow) and adjacent active extravasation (arrowhead).

**Figure 3:**
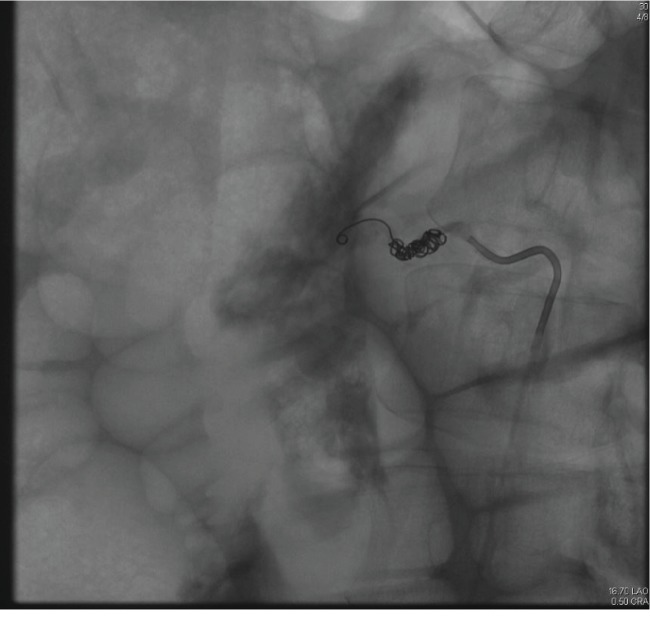
Postembolization image demonstrates multiple metallic coils and cessation of flow within the right ovarian artery.

The patient was taken directly to interventional radiology, where an abdominal aortogram showed an area of contrast extravasation in the right upper quadrant. Selective right renal and right upper lumbar injections showed no abnormalities. The right ovarian artery was cannulated, and injection showed a pseudoaneurysm of the proximal right ovarian artery with associated active extravasation of contrast (Figure 2). A 3 fr coaxial Progreat catheter (Terumo) was advanced through the 5 fr angiographic catheter, and the vessel was embolized to the point of occlusion with multiple metallic microcoils measuring 3 to 5 mm diameter (Figure 3).

The patient received an additional transfusion of 3 units packed RBCs the following day. The patient's clinical course stabilized, and she was discharged seven days after admission. The final hemoglobin/hematocrit rose to 8.7/24.9, the WBC count decreased to 17.5, and BUN/creatinine decreased to 13/1.4. The remote history of chronic lymphocytic leukemia was not considered a factor in the events of the current admission.

## 3. Discussion

Ovarian artery aneurysms are relatively rare lesions, and the majority are related to pregnancy, either late in its course or in the early postpregnancy period [1-4]. Several factors have been implicated, including hemodynamic changes such as increased blood volume and cardiac output as well as hormonal changes predisposing to aneurysm formation [1–4]. We have been able to find only seven reported cases of ovarian artery pseudoaneurysms in postmenopausal patients [2–8]. Two additional cases were reported in females of age 44 and 48, the first two years after a cesarean section [9, 10].

There had been an indication for surgical intervention in the remote past, but all reported cases in recent history have been successfully managed with transcatheter embolization. The case we present is an additional rare case of spontaneous retroperitoneal hemorrhage related to an atraumatic rupture of an ovarian artery pseudoaneurysm in a postmenopausal woman successfully managed with transcatheter embolization.
